# Anoikis in cell fate, physiopathology, and therapeutic interventions

**DOI:** 10.1002/mco2.718

**Published:** 2024-09-15

**Authors:** Jie Mei, Xue‐Yao Jiang, Hui‐Xiang Tian, Ding‐Chao Rong, Jia‐Nan Song, Luozixian Wang, Yuan‐Shen Chen, Raymond C. B. Wong, Cheng‐Xian Guo, Lian‐Sheng Wang, Lei‐Yun Wang, Peng‐Yuan Wang, Ji‐Ye Yin

**Affiliations:** ^1^ Department of Clinical Pharmacology Xiangya Hospital, Central South University Changsha Hunan China; ^2^ Institute of Clinical Pharmacology Hunan Key Laboratory of Pharmacogenetics Central South University Changsha Hunan China; ^3^ Engineering Research Center of Applied Technology of Pharmacogenomics Ministry of Education Changsha Hunan China; ^4^ National Clinical Research Center for Geriatric Disorders Xiangya Hospital, Central South University Changsha Hunan China; ^5^ Oujiang Laboratory Key Laboratory of Alzheimer's Disease of Zhejiang Province Institute of Aging Wenzhou Medical University Wenzhou Zhejiang China; ^6^ School of Life Sciences Westlake University Hangzhou Zhejiang China; ^7^ Centre for Eye Research Australia Royal Victorian Eye and Ear Hospital Melbourne Victoria Australia; ^8^ Ophthalmology Department of Surgery The University of Melbourne Melbourne Victoria Australia; ^9^ Center of Clinical Pharmacology the Third Xiangya Hospital Central South University Changsha Hunan China; ^10^ Department of Pharmacy Traditional Chinese and Western Medicine Hospital of Wuhan, Tongji Medical College, Huazhong University of Science and Technology Wuhan Hubei Province China

**Keywords:** anoikis, cell death, circulating tumor cells, extracellular matrix, integrin, tumor metastasis

## Abstract

The extracellular matrix (ECM) governs a wide spectrum of cellular fate processes, with a particular emphasis on anoikis, an integrin‐dependent form of cell death. Currently, anoikis is defined as an intrinsic apoptosis. In contrast to traditional apoptosis and necroptosis, integrin correlates ECM signaling with intracellular signaling cascades, describing the full process of anoikis. However, anoikis is frequently overlooked in physiological and pathological processes as well as traditional in vitro research models. In this review, we summarized the role of anoikis in physiological and pathological processes, spanning embryonic development, organ development, tissue repair, inflammatory responses, cardiovascular diseases, tumor metastasis, and so on. Similarly, in the realm of stem cell research focused on the functional evolution of cells, anoikis offers a potential solution to various challenges, including in vitro cell culture models, stem cell therapy, cell transplantation, and engineering applications, which are largely based on the regulation of cell fate by anoikis. More importantly, the regulatory mechanisms of anoikis based on molecular processes and ECM signaling will provide new strategies for therapeutic interventions (drug therapy and cell‐based therapy) in disease. In summary, this review provides a systematic elaboration of anoikis, thus shedding light on its future research.

## Graphical abstract

1. Anoikis is an integrin‐dependent form of apoptosis and can affect cell fate.

2. Anoikis widely exists in the physiological and pathological processes of life.

3. Anoikis‐based regulatory strategies are potential targets for therapeutic intervention.

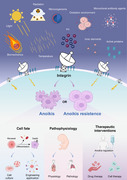

## 1 INTRODUCTION

In the process of tissue and cell growth and differentiation, the factors that determine cell fate and tissue structure are not only the regulation of internal gene inheritance, but also the external environment. Among them, the extracellular matrix (ECM), which comprises at least a third of tissue structures, take a significant role in the localization,^1^ function,^2^ and fate of cells.^3^ Most of the cells that constitute tissues and organs in living organisms need a specific anchoring environment to survive,^4^ which is usually specifically encoded by individual genetic development patterns.^5^ If cells fail to maintain proper ECM attachment, anoikis, a programmed autonomously regulated mechanism, is triggered to initiate cell death and thus maintain tissue homeostasis.^6^ Anoikis was first discovered in 1994 by disrupting the interaction of normal epithelial cells and ECM.^7^ Early researchers believed that anoikis is a programmed cell death triggered by cell detachment from ECM, which avoids distant organ colonization by preventing nonadherent cell growth and improper cell adhesion.^8^, ^9^ In‐depth research reveals integrin's crucial role in the occurrence of anoikis.^10^ Therefore, the Nomenclature Committee on Cell Death defined anoikis as a specific form of intrinsic apoptosis triggered by integrin‐dependent anchorage deficiency.^11^

When anoikis occurs, integrin, a heterodimeric transmembrane protein composed of α and β subunits,^12^ which is a key dependent component of ECM signaling, not only provides a physical connection to the cytoskeleton,^13^ but also transduces ECM signaling to the cell.^13^, ^14^ Some studies have proved that integrin induces cell anoikis through cell cycle arrest,^15^, ^16^ cytoskeleton changes,^17^ and loss of cell adhesion function.^18^ Integrins serve as primary transmembrane receptors for cell adhesion, transmitting mechanical signals and regulating physiological and biochemical behaviors in response to the ECM^19^; therefore, they are seen as essential members of anoikis.^10^ As a family of transmembrane receptors mediating physical and chemical signaling between cells and the ECM,^20^ integrins are expressed in a wide range of cells.^21^, ^22^ Because of its adhesion effect, integrins often have a significant impact on cell anchor‐dependent growth,^23^ and therefore regulate cell fate.^24^ Extracellular signals include chemical signals, such as monoclonal antibody agents (abciximab, natalizumab, vedolizumab),^25^ active proteins (fibronectin, osteopontin),^26^, ^27^ divalent cations,^28^ oxidation environment,^29^ as well as physical signals, such as biomechanics (shear stress, stiffness),^30^, ^31^ magnetic field (radiation),^32^ pH,^33^, ^34^ light,^35^ temperature,^36^ can disrupt integrin ligation, resulting from impaired cell adhesion adaptability and leading to the occurrence of anoikis.

**FIGURE 1:**
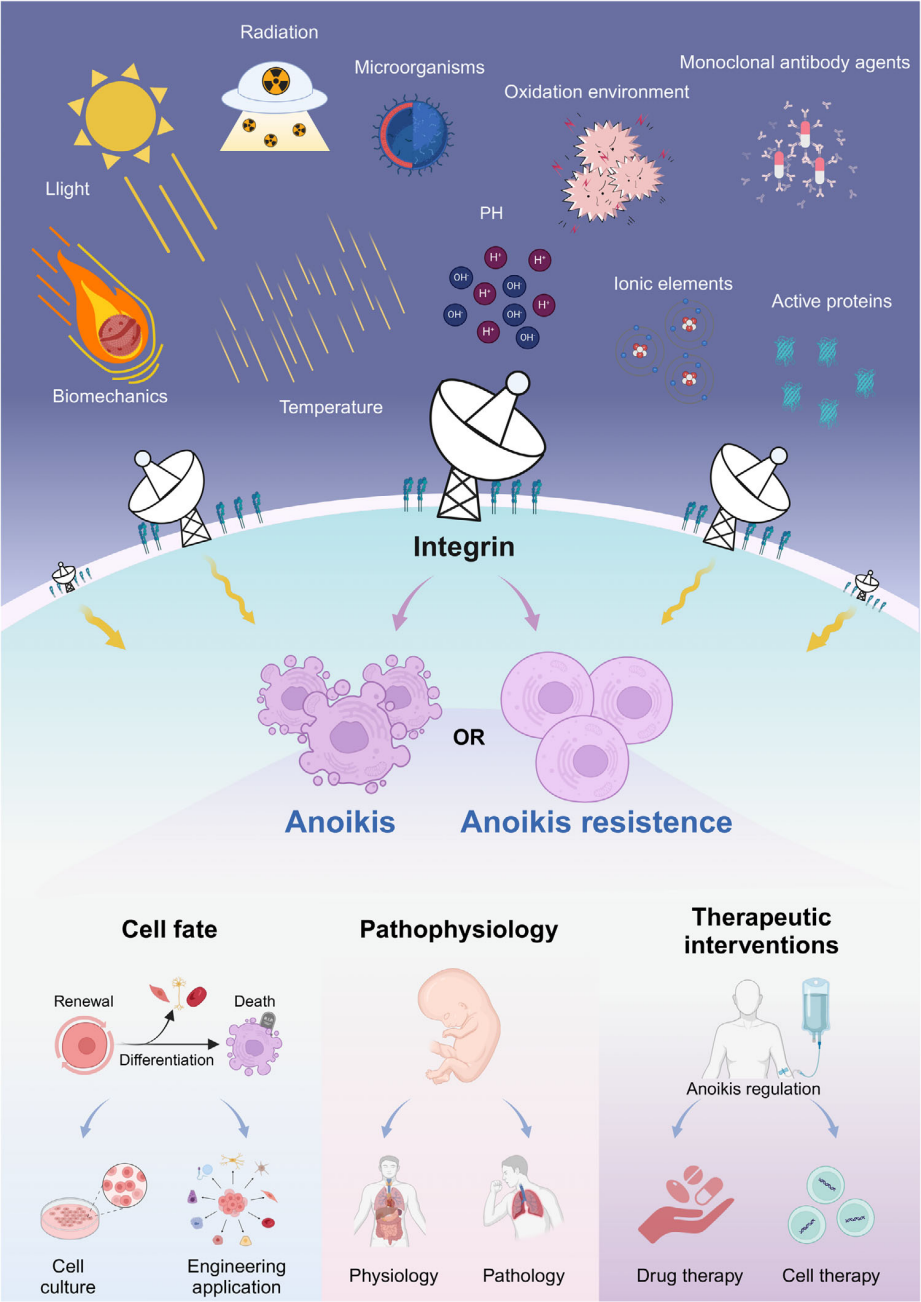
Extracellular signal regulation and transformation applications of anoikis. Integrin transmits ECM signals including physical and chemical signals to regulate anoikis, thereby regulating cell fate and participating in physiopathological processes of the body, which is of great significance for therapeutic interventions.

Based on integrin‐mediated cell adhesion adaptation, external adaptive environmental changes would imply that anoikis has participated in extensive biological processes, such as cell fate (renewal, differentiation, cell death, etc.)^37^ and physiopathology (embryonic development, tissue repair, disease occurrence, etc.).^38^ In addition, in oncology studies, anoikis has been involved in malignant transformation of tumors,^39^ remote colonization of circulating tumor cells (CTCs),^40^ regulation of immune environment,^41^ response to drug therapy,^42^ and prediction and prognosis of diseases.^43^ This means that regulatory strategies based on anoikis will be promising targets for disease treatment and interventions, such as regulating anoikis to affect the drug efficacy,^44^ tumor metastasis,^45^ tumor stemness,^46^ and the activity of engineered stem cells.^47^ In particular, in stem cell and engineering transformation researches, anoikis poses new challenges to the in vitro and in vivo application of embryonic stem cells (ESCs), induced pluripotent stem cells (iPSCs) and other engineering cells.^47^ Therefore, this review introduces the research progress of anoikis and its main molecular processes, focusing on its role in cell fate, physiopathological regulation, and emphasizes the potential value of anoikis‐based therapeutic intervention strategies in disease (Figure 1). We also raise the question of anoikis’ in‐depth thinking and new insights into its potential research directions.

## 2 MOLECULAR PROCESS OF ANOIKIS

Notably, anoikis is defined as a broad and complex regulatory mechanism to specifically refer to a mode of apoptosis induced by ECM alterations in response to extracellular physical or chemical signals. Considering the clear signaling flowthrough involved, anoikis concatenates specific triggers and cascading outcomes, which provides a more comprehensive understanding of how cell respond to extracellular stimuli and how to transfer this stimulus to downstream pathways to induce apoptosis. From the perspective of incoming signals of anoikis, physical stimulus, such as hyperthermia, which affects the integrin cytoskeleton network, have the potential to influence the cell morphology and further leads to anoikis,^48^ and shear stress, which modulates integrin β1 adhesion kinase‐mediated multicellular aggregation, thus is able to regulate anoikis resistance.^31^ Additionally, chemical signals may also lead to anoikis. For example, an altered fibronectin matrix regulated integrin v‐mediated FAK and ERK phosphorylation, leading to anoikis.^26^ Currently anoikis is thought to be mainly mediated by membrane adhesion signaling molecules (integrin family proteins), thus anoikis can respond to both physical and chemical stimuli and may cross‐talk the same downstream signaling pathways via integrin, implying its possible integral role in cell fate regulation, especially cross‐talking physical and chemical signals.

**FIGURE 2:**
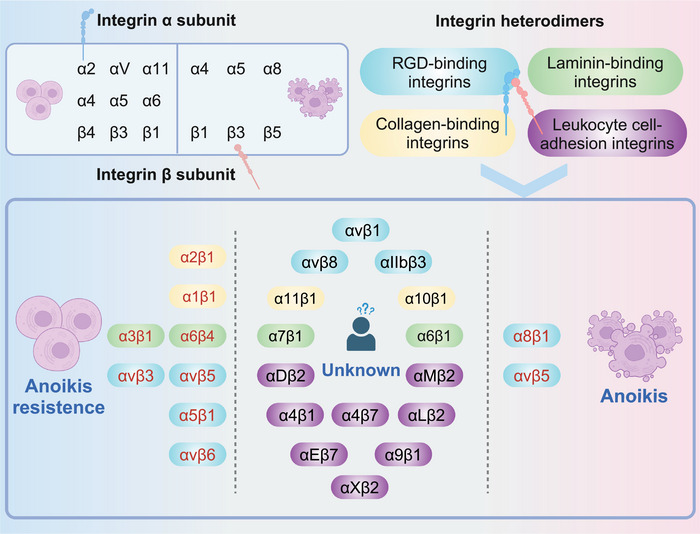
The regulatory relationship between the integrin family and anoikis. The results and mechanisms of different integrin subunits and their dimers regulating anoikis are different.

The currently documented integrin family consisted of 18 α subunits and 9 β subunits,^49^ as well as the recently reported integrin subunit β‐like 1 (ITGBL1),^50^ and different α and β subunits have similar structural characteristics^51^, ^52^ (Table 1). The interaction between different subunits forms 24 different heterodimers, including the Arg‐Gly‐Asp (RGD)‐binding integrins (αvβ1, αvβ3, αvβ5, αvβ6, αvβ8, α8β1, α5β1, and αIIbβ3), leukocyte adhesion integrins (α4β1, α9β1, αLβ2, αMβ2, αXβ2, αDβ2, α4β7, and αEβ7), collagen‐binding integrins (α1β1, α2β1, α10β1, and α11β1), and laminin‐binding integrins (α3β1, α6β1, α7β1, and α6β4).^53^ Although anoikis was reported as a integrin‐dependent pathway, however, the exact mechanisms underlying their interrelationships remain largely unclear^11^ (Figure 2). Years ago, anoikis studies focused on individual subunits. For example, the ITGA5,^54^ ITGA6,^55^ and ITGAV^56^ out of the α subunit family were reported to be associated with the anoikis resistance. However, the understanding of mechanisms underlying the interactions between individual integrin subunits and anoikis was challenged by conflicting findings such as the recent report of ITGB5 in promoting anoikis.^57^ Meanwhile, investigations into the β subunits found that ITGB4 could inhibit anoikis,^58^ ITGB5 promoted anoikis,^47^ while ITGB1^59^, ^60^ and ITGB3^61^, ^62^ showed a multidirectional regulation of anoikis. Therefore, more and more recent studies started to investigate the function of diverse integrin heterodimers in anoikis. For example, the collagen‐binding integrins (α1β1, α2β1)^63^, ^64^ and laminin‐binding integrins (α3β1 and α6β4)^65^, ^66^ show consistent inhibitory effects on anoikis. Interestingly, the RGD‐binding integrin family showed bi‐directional functions, including that αvβ5,^67^ αvβ6,^68^ and α5β1^65^ demonstrate regulatory effects inhibiting anoikis, whereas αvβ3^69^ and α8β1^70^ have been reported to exhibit promoting effects. However, few correlations between leukocyte adhesion integrin family and anoikis have been reported. Although we have expanded our understanding of the interrelationship between integrins and anoikis by investigating the function of specific heterodimers, however, it seems that their underlying mechanisms were quite complex as there were still some contradictory findings like the αvβ3 may also have an inhibitory role in anoikis.^71^, ^72^ Therefore, further research is essential to explore the cellular distribution of integrins and the cross‐talk between integrin families.^73^, ^74^ This exploration holds the potential to contribute to a more nuanced understanding of the intricacies involved in the process of integrin‐mediated anoikis.

**TABLE 1:** The detail of the integrin subunits.

Gene name	Protein (UniProt)	Structure	Gene name	Protein (UniProt)	Structure
Integrin α subunit					
ITGA1	Integrin alpha‐1 (P56199)	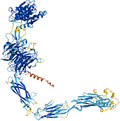	ITGA2	Integrin alpha‐2 (P17301)	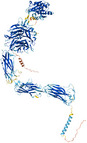
ITGA2B	Integrin alpha‐IIb (P08514)	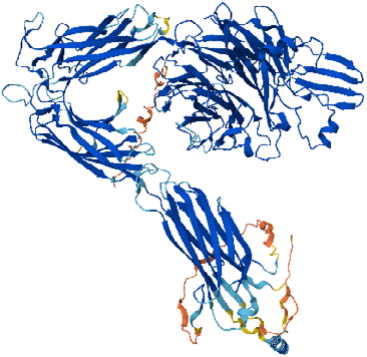	ITGA3	Integrin alpha‐3 (P26006)	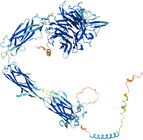
ITGA4	Integrin alpha‐4 (P13612)	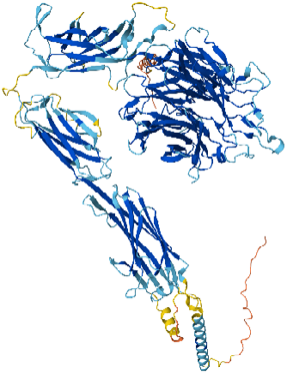	ITGA5	Integrin alpha‐5 (P08648)	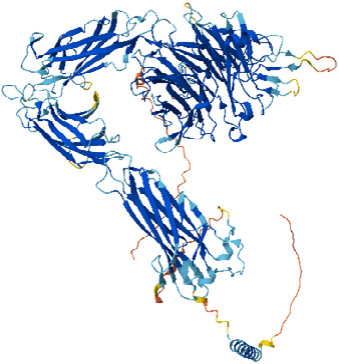
ITGA6	Integrin alpha‐6 (P23229)	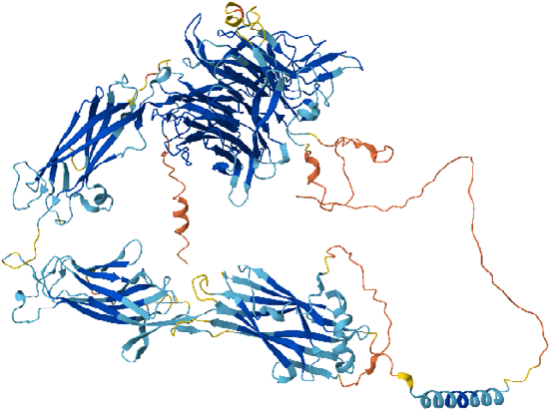	ITGA7	Integrin alpha‐7 (Q13683)	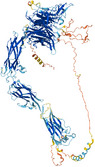
ITGA8	Integrin alpha‐8 (P53708)	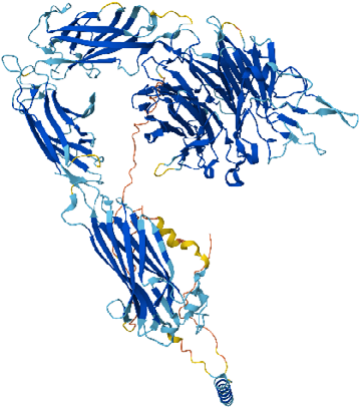	ITGA9	Integrin alpha‐9 (Q13797)	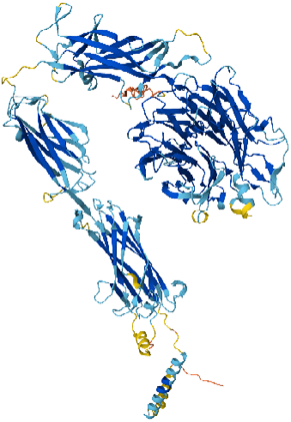
ITGA10	Integrin alpha‐10 (O75578)	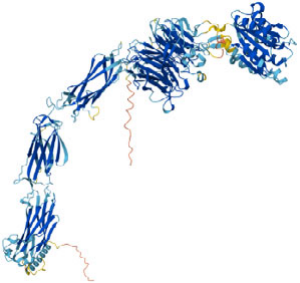	ITGA11	Integrin alpha‐11 (Q9UKX5)	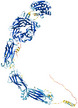
ITGAD	Integrin alpha‐D (Q13349)	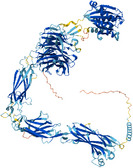	ITGAE	Integrin alpha‐E (P38570)	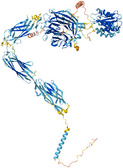
ITGAL	Integrin alpha‐L (P20701)	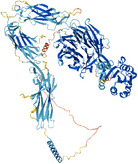	ITGAM	Integrin alpha‐M (P11215)	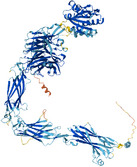
ITGAV	Integrin alpha‐V (P06756)	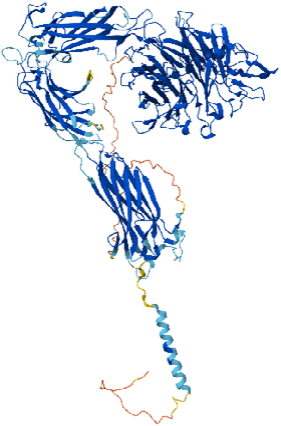	ITGAX	Integrin alpha‐X (P20702)	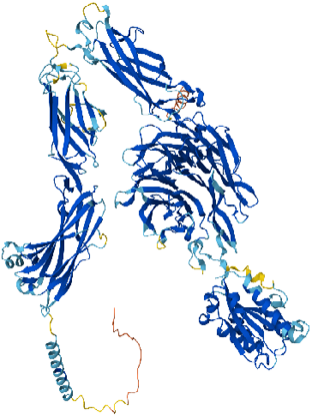
Integrin β subunit					
ITGB1	Integrin beta‐1 (P05556)	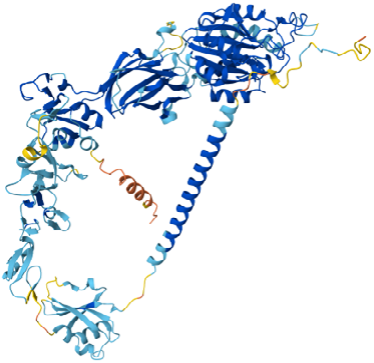	ITGB2	Integrin beta‐2 (P05107)	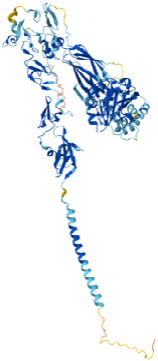
ITGB3	Integrin beta‐3 (P05106)	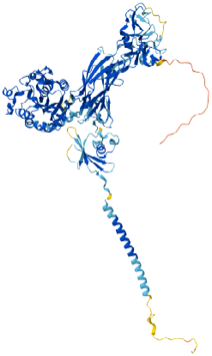	ITGB4	Integrin beta‐4 (P16144)	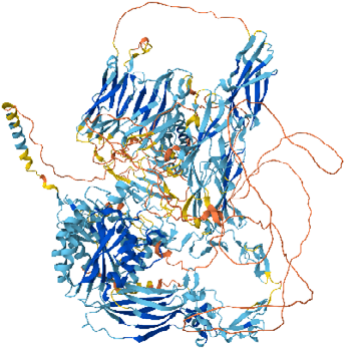
ITGB5	Integrin beta‐5 (P18084)	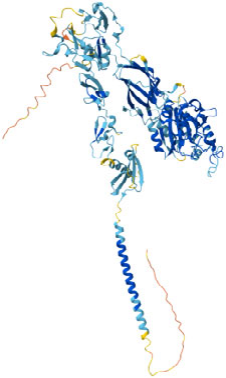	ITGB6	Integrin beta‐6 (P18564)	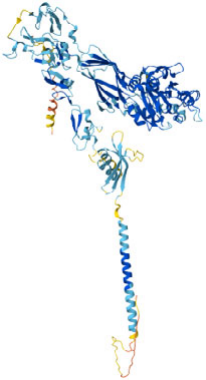
ITGB7	Integrin beta‐7 (P26010)	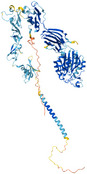	ITGB8	Integrin beta‐8 (P26012)	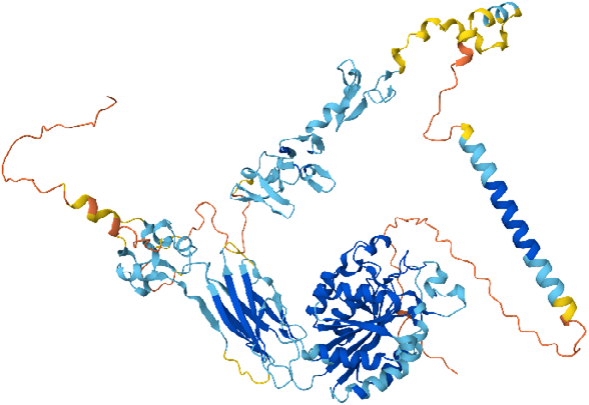
ITGBL1	Integrin beta‐like protein 1 (O95965)	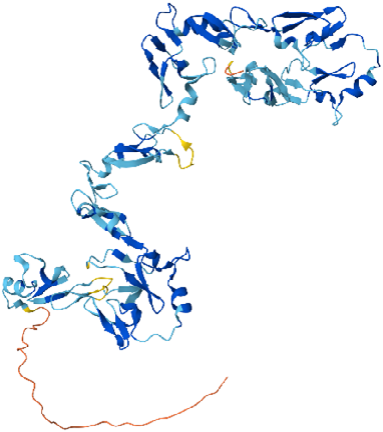			

The structure information of α and β subunits of integrin were obtained from AlphaFold Protein Structure Database (https://alphafold.ebi.ac.uk/)

In the context of intracellular regulatory processes, downstream of the ECM interactions are the apoptosis mechanisms triggered by the anoikis as feedback of extracellular changes, which can be further divided into intrinsic pathways or extrinsic pathways. Specifically, intrinsic pathways are often associated with cell apoptosis trigged by mitochondrial disturbance, while extrinsic pathways are normally activated by cell death receptors involved in the anoikis.^75^ On the one hand, a typical intrinsic pathway downstream of the detachment between the ECM and integrin‐mediated adhesion is the phosphorylation of survival effectors, such as the well‐known FAK and PI3K/Akt pathways.^76^ Apoptotic protein family members, such as BCL‐2, can be suppressed by the FAK phosphorylation, promoting the activation of apoptotic Bax and Bak, which can move from cytoplasmic ectopia to mitochondrial outer membrane to form oligomers, causing mitochondrial membrane permeability and cytochrome c release through the formation of small pores.^77^ Meanwhile, the phosphorylated FAK pathway suppresses the activity of its downstream PI3K/Akt pathway, resulting in the release of cytochrome c, caspase‐9, and the auxin apoptotic activator to form an “apoptotic body (disc)” that activates the downstream caspase‐3 to perform the apoptotic process.^78^ On the other hand, extrinsic pathways recruit cell death receptors, including the Fas receptor, and the tumor necrosis factor‐α, to activate cell apoptosis.^79^ For example, the Fas receptor forms the death inducible signal complex (disc) with its ligand FasL, recruiting the caspase‐3, ‐6, and ‐7 for cell apoptosis.^80^ The activation of the extrinsic pathways may follow some types of mitochondrial damages, implying the potential cross‐talk between the intrinsic and extrinsic pathways induced by the anoikis.^81^ In summary, the anoikis information flow starts from the ECM changes induced by physical or chemical signals, though the role of integrins in transferring these signals was largely unclear, end in the activation of cell apoptosis pathways which majorly rely on intrinsic mitochondrial disturbance or cell apoptosis processes mediated by the activation of the extrinsic cell death receptors. Therefore, the anoikis plays a significant role in cell fate regulation and recruits a large number of molecules which play different roles in different life activities to respond to extracellular changes.

## 3 ANOIKIS IN CELL FATE

To explore the role of anoikis in cell fate, researchers focused on the role of integrin‐mediated ECM signaling regulation mechanism of anoikis in in vitro research models. Currently, many in vitro models, such as cell models, organoid models, and animal models, were developed to simulate the original cell fate process and physiological environment, providing convincing and comprehensive evidence for pathology and pharmacology discoveries.^82^ For example, tumor research has been boosted by the establishment of human tumor cell lines in vitro, starting from the first cultured HeLa cells^83^ in 1950s to primary cells,^84^ and more recent engineered cells.^85^ However, how these ECM alterations or in vitro environment changes affect or promote the establishment of in vitro models was largely debated. The potential of anoikis in responding to ECM changes suggests that its role in these models may largely overlooked currently.

### 3.1 Anoikis in cell culture

The traditional 2D model used to dominate the realm of in vitro culture for a long time.^86^, ^87^ Presently, common vessels for 2D adherent cell culture encompass flat polymethylmethacrylate coatings, cell culture glass slides, and tissue culture plates (TCPs), among others. However, an interesting truthy is that even the same type of cells often behaves differently, such as performing distinct growth phenotypes, when culture on different material surfaces.^88^ This means that different cell contact surfaces, or cell culture planes, have an impact on cell growth and life processes. In other words, the way that cells respond differently to adhesion of culture surfaces thereby resulting in different cell fates.^89^, ^90^ For example, to address the in vitro survival of primary intestinal epithelial cells, collagen‐ordered membrane schemes were designed to increase the survival of intestinal cells and helped to maintain their function.^91^ As an anchorage‐dependent mode of programmed death regulated by ECM signaling,^92^ anoikis is often directly involved in culture planes‐mediated cell fate processes. Years ago, researchers have found on some nonintegrin‐recognizable matrices, such as poly(N‐p‐vinylbenzyl‐4‐O‐beta‐d‐galactopyranosyl‐d‐gluconamide) and poly‐l‐lysine, that hepatocytes (adherent‐dependent cells) developed anoikis in response to ECM disruption.^93^ In addition, silicon‐substituted hydroxyapatites, a class of biomaterials different from traditional two‐dimensional cultures materials, have been reported to induce cell anoikis by affecting the signaling at the interface between cells and biomaterials and altering cell adhesion, which helped to delay osteoblast differentiation and had potential applications for therapies such as osteoporosis.^94^ On the contrary, collagen coatings of interconnected macroporous nanometric carbonated hydroxyapatite/agarose scaffolds were proved to have an inhibitory effect on anoikis,^95^ implying that by influencing the interaction of cell culture planes with cells could be one of the means to modulate the cell fate of anoikis.

In 3D cell culture, it is considered to be a more appropriate way to regulate cell fate for in vitro cell modeling. A recent study compared the melanoma cells cultured in 2D and 3D environments, revealing substantial changes in cell adhesion‐related signaling pathways and anoikis resistance related gene expressions.^96^ This means that unlike the traditional 2D culture modes, 3D culture will bring about new microscopic changes to the cell culture modes. At present, more 3D culture approaches, including spheroids,^97^, ^98^ organoids,^99^ microcarriers,^100^ and microgravity,^101^ have altered cell fate to some extent, especially the regulation of anoikis. With respect to the classical ovarian cancer metastasis models, it has been clarified that tumor cells formed 3D structures such as spheroids in order to survive in the nonadherent state to resist anoikis and thus gained the survival power for distal metastasis,^102^, ^103^ in which integrin αvß3‐mediated cell adhesion process may be the key mechanism of anoikis resistance.^104^ Thus, some derived nonadherent or ultra‐low adhesion 3D culture modes are widely used in sphere formation assay, assessment of tumor malignancy or stemness due to their unique anchoring‐independent growth or anoikis resistance.^105^ Moreover, researchers have long found that the contact surface between the cells and the culture system requires a certain connection or else there will be attrition of the cells.^106^ Therefore, the injectable cell‐laden microcarriers, a class of suspensions in hydrogels, were developed to help the cells to undergo adhesive growth thereby avoiding anchorage‐dependent cell death, which had a positive significance in addressing cell loss during osteogenic differentiation of mesenchymal stem cells (as representative anchorage‐dependent cells).^107^ And it was found that the microcarriers can help the cellular integrins and extracellular ligands to connect to achieve stable anchorage‐dependent cell growth, which is largely attributed to the promotion of anoikis resistance.

With the establishment of common understandings about the influence of the ECM alterations on cell fate, more and more studies started to consider the interactions between cells and its microenvironments, which in turn to help directing novel culture techniques to mimic a in vivo microenvironment to promote cell culture better.^108^, ^109^ It also enhances our comprehension of intricate cell–cell and cell–matrix interactions, ultimately paving the way for the establishment of more advanced research systems. As a result, subsequent strategies, including suspension culture,^110^, ^111^ coculture methodologies,^112^ 3D cultivation,^113^ specialized biomaterial‐based cultivation,^88^, ^114^ and in vivo culture systems,^115^ were developed towards creating more suitable ECM conditions to realize more precise regulation of cell fate during in vitro cell culture.

However, it is widely accepted that current preclinical models, especially in vitro cell models, are hard to accurately replicate the in vivo cellular microenvironment, biological intricacies and biophysical factors, leading to the potential impacts on drug sensitivity testing. By contrast, 3D cultivation systems were thought to be a better mimic of in vivo conditions, which enhances their sensitivity in drug screening.^116^ This phenomenon may be attributed to adopting rounded morphologies and forming small hemispherical plasma membrane protrusions of cells to promote the creation of signaling hubs proximal to the plasma membrane, which often leads to anoikis resistance that facilitates a better survival rate facing ECM alterations.^117^ Given the pivotal role of anoikis resistance in tumor migration and metastasis, and recognizing that cell aggregation is an important mechanism for conferring anoikis resistance,^118^ material scientists developed a similar in vitro platform by modifying TCPs to create a fluoro‐silica surface to finely regulate cell aggregation–disaggregation events, further enhancing our comprehension of intricate cell–cell and cell–matrix interactions.^119^

In summary, learned from the development of cell culture systems, it is essential to highlight that factors including genetic regulations, chemical interventions, and physical environmental stimulus, could fundamentally lead to different cell fate, and in particular, different response mechanisms to anoikis. Therefore, considering the key role of anoikis in responding to these factors, mechanisms underlying anoikis resistance have the potential to direct novel strategies for in vitro cell culture. However, the optimal ECM culture environments vary a lot for various cell types, leading to the difficulty in determining a universal system to facilitate the appropriate physiological reflections of various cell fate in vivo. In summary, knowledge about anoikis is the powerful tool for developing promising cell culture systems to simulate in vivo conditions as much as possible.

### 3.2 Anoikis in engineering applications for stem cells

However, for engineered cells such as iPSCs, the focus is not on simulating the state and characteristics of cells in vivo, but on exploring new directions for cell function.^120^ Certainly, engineering cells including ESCs,^121^ mesenchymal stem cells,^122^ hematopoietic stem cells,^123^, ^124^ and neural stem cells^125^, ^126^ are not excluded as suitable tools for simulating cell lines in vivo. Importantly, as vital models for studying cell fate and unknown functions, they eliminate the challenges of exploring the primitive ECM. Then, the research on the cell death mechanism including anoikis and its engineering transformation value will be purer.

Since their derivation, stem cells such as human embryonic stem cells (hESCs) have been used in various studies, including developmental biology, pathology, and genomics.^127^, ^128^ However, hESCs are subjected to constant anoikis in cell culture,^129^ as evidenced by the fact that one of the main difficulties in hESCs culture is that they are particularly sensitive to dissociation, which is precisely the operative process underlying cell culture in vitro.^130^ Considering that hESCs is highly susceptible to cell death after dissociation into single cells, researchers have long believed that cell adhesion signal perception and anoikis caused by cell detachment may be important incentives.^131^ In stem cell‐derived cell lines, such as hESCs‐derived retinal pigment epithelial (hESC‐RPE), it is also widely found that ECM detachment, serum deprivation, hydrogen peroxide stimulation can induce anoikis, which undoubtedly restricts the research of such stem cells in the pathogenesis of age‐related macular degeneration, stem cell therapy in vivo, and other fields.^132^

On the other hand, the cryopreservation of hESCs still have the problems of low recovery rate and loss of pluripotency after thawing.^133^ Although the transcriptome data of the cells after thawing showed relative stability, the expression of genes related to cell morphology, death, regeneration, and differentiation has changed. Researchers have pointed out that anoikis is a promising target for improving the survival rate and maintaining pluripotency of hESCs after cryopreservation.^133^ As a promising source of cell therapy for retinal degenerative diseases, hESC‐RPE is also facing the same issues of timeliness and functionality of cryopreservation. It has been found that hESC‐RPE cryopreserved at passage 2 day 5 tends to maintain a certain high quality of cell viability and adhesion.^134^ As for human iPSC (hiPSC), which is the most promising stem cell therapy, researchers have begun to pay attention to the effect of cryopreservation on the activity of hiPSC.^135^ hiPSC‐derived retinal pigment epithelium (hiPSC‐RPE) cells have been explored the effects of cell activity and metabolic functions at different freezing temperatures and was found that cell viability is correlated with preservation time and temperature, with the best cell status at 16°C and a significant reduction in anoikis.^136^ Therefore, for stem cell research, simple and efficient cryopreservation methods are urgently needed to maintain cell activity and function. The mechanism by which temperature affects cell cryopreservation and resuscitation activity is poorly understood and may involve alterations in cell matrix adhesion and secretory protein activity due to changes in the elastic modulus of the ECM.^137^ But as an anoikis‐dependent pathway, the role of integrin signaling in hESCs maintenance is highly promising.

Stem cells such as iPSCs are also continuously affected by anoikis in culture,^129^ which are also prone to anoikis after single cell dissociation.^138^ And anoikis serves as the primary factor contributing to low survival rates following transplantation and represents a significant hurdle in the field of stem cell therapy.^139^, ^140^ Cell therapy based on neural stem cells is faced with the problem of cell transplantation‐related survival and fate regulation. Most of the implanted cells will die due to oxidative stress, anoikis and other factors within few days after transplantation. Interestingly, cell aggregates, such as neurospheres, can prevent anoikis and increase cell viability by providing a special extracellular support environment, and the effect of colony sphere size on this process is important.^141^ Mesenchymal stem cells with multilineage differentiation ability also showed sensitivity to ECM adhesion. It has been reported that miR‐125b, an adhesion‐regulated microRNA, can protect mesenchymal stem cells from anoikis.^47^ In addition, regulation of ITGA5B1 expression can also increase the cell adhesion, cell viability of bone marrow mesenchymal stem cells by inhibiting cell anoikis,^142^ which is of great reference significance for stem cell therapy. Anoikis is also a rate‐limiting factor in the formation of cardiomyocytes in the heart transplantation therapy of hESC‐derived cardiomyocytes (hESC‐CMs).^143^ Currently, some superior therapies for cotransplantation of biocompatible materials with hESC‐CMs and promoting heart regeneration have been reported. This may be the result of providing unique ECM support to overcome anoikis.^144^

**FIGURE 3:**
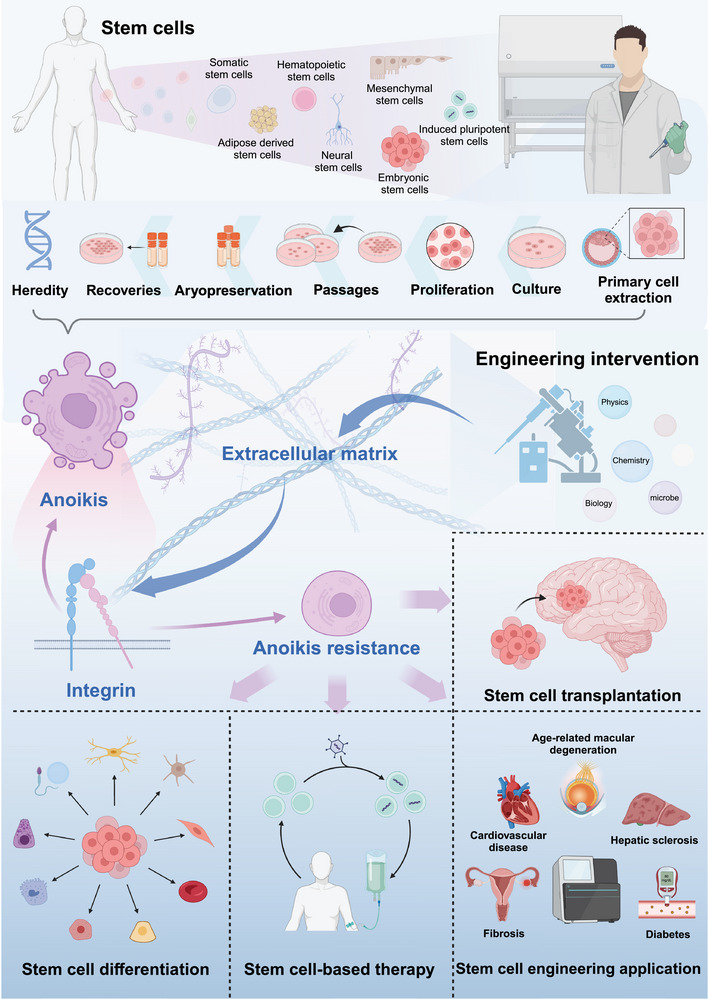
Engineering applications of anoikis. Conventional cell in vitro culture systems, including primary cell extraction, culture, proliferation, passage, cryopreservation, recovery, and heredity, continue to be blocked by anoikis. Physical, chemical, and biological regulatory processes based on ECM can regulate cell fate by affecting integrin, which is of great value for stem cell‐based differentiation, transplantation, stem cell therapy, and other engineering applications.

As a process in which cells sense extracellular signals and directly transmitted intracellular regulation of cell death fate, anoikis undoubtedly proposes a potential direction for the culture and research transformation of engineering cells. The integrin‐dependent mechanism based on anoikis implies that integrin, as a cell adhesion and extracellular mechanoreceptor,^145^ is an important anoikis regulatory pathway. Integrin can sense the mechanical force generated by the matrix, thereby converting these stimuli into downstream signals that regulate cell viability.^146^ Therefore, anoikis will be regulated when engineering cells perform extracellular signal regulation, including physical and mechanical, chemical, and other signal stimulation. For example, molecular inhibitors, special petri dish surface topologies and some biological culture materials influenced the cell survival by affecting integrin on the cell surface.^59^, ^93^, ^147^ This is certainly a promising direction for researchers wishing to study the efficient culture of stem cells in vitro, iPSC differentiation, stem cell transplantation, stem cell therapy, and other engineering applications (Figure 3).

## 4 ANOIKIS IN PHYSIOPATHOLOGY

**FIGURE 4:**
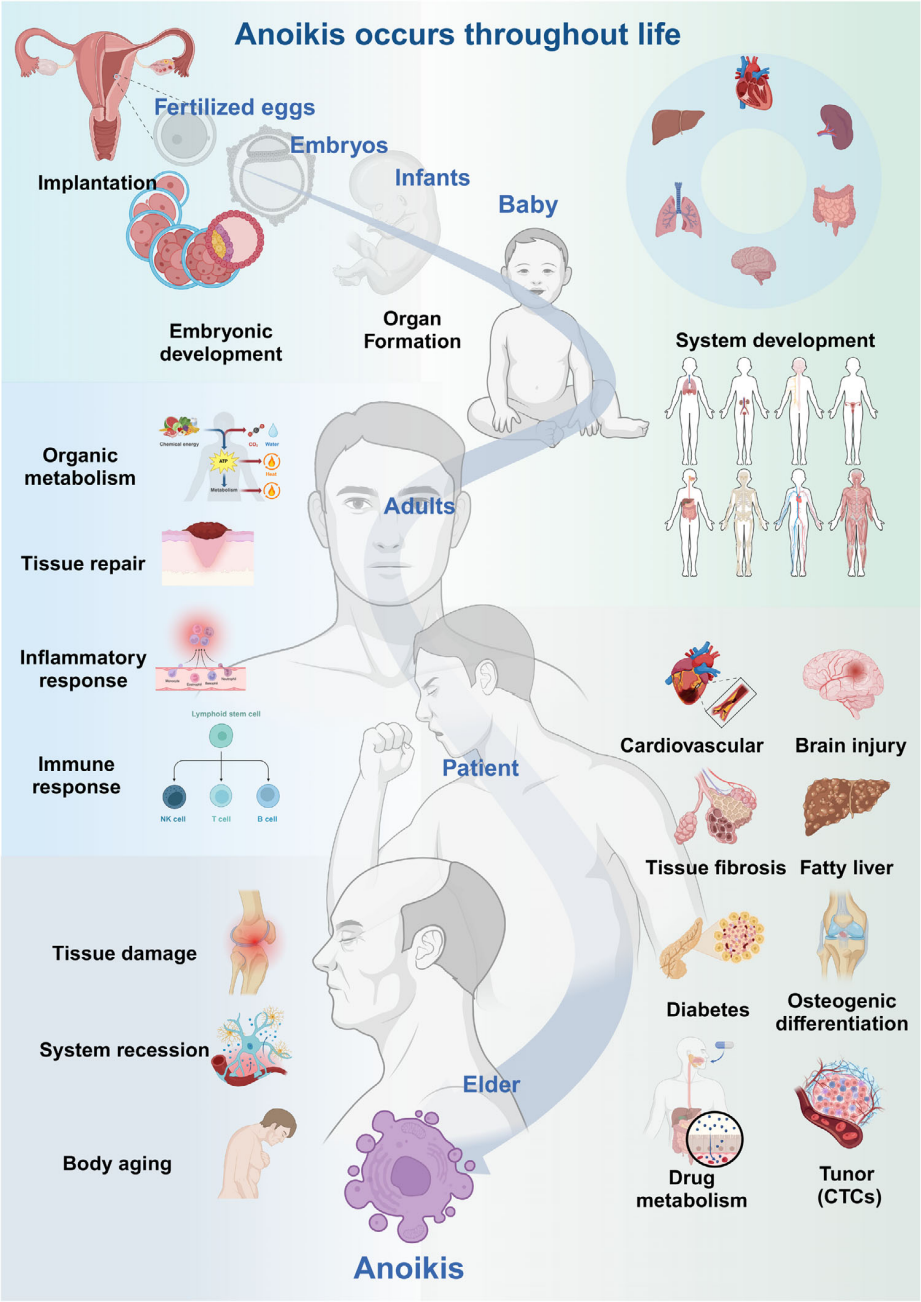
Anoikis are involved throughout the life. Anoikis is involved in the process from implantation of the fertilized egg to embryonic development, tissues and organs development, homeostasis maintenance, pathological processes, and body aging. CTCs, circulating tumor cells.

Cell death serves as the ultimate fate of cells, representing not merely an endpoint, but rather a protracted process.^148^ Similarly, when cells encounter an unsuitable ECM environment, their surface integrin‐mediated connections are disrupted, inciting the activation of anoikis signals, following a long cascade to regulate physiological activities of cells in respond to the disruptions.^149^ Accompanied by the cell self‐rescue mechanism, an intricate cellular fate‐regulating process ensues within the extensive cell populations, influencing the overarching biological functions of the organism as a whole. Consequently, anoikis functions not only as a regulator of cellular fate, but also as a vital driver for the sustenance of individual life (Figure 4).

### 4.1 Anoikis in physiology

Life begins from the implantation of the fertilized egg in the uterus.^150^ During the beginning stage after implantation, the fertilized egg need to adapt the diverse ECM in the uterus to start cell division and differentiation, and quickly establish the embryonic development.^151^, ^152^ Studies have shown that stratified epithelial budding, as the first step of branching morphogenesis in embryonic organs undergoing a critical stage of epithelial morphogenesis, is driven by an overall combination of strong cell–matrix adhesion and weak cell–cell adhesion of peripheral epithelial cells. And in‐depth studies have found that β1‐integrin‐mediated cell–matrix adhesion was required for successful budding,^153^ implying that integrin‐mediated cell adhesion and anoikis have been involved in the physiological processes of life since early embryonic life. Later on, the fetus undergoes a phase of rapid growth and accelerated metabolism before the age of one,^154^ bringing the challenge about overcoming dynamical ECMs and continue growing robustly. Especially, how these dynamically changing cells, such as neurons, bone, and muscle cells, overcome the challenges posed by an unsuitable ECM and continue to grow robustly remains a question that requires further exploration.^155^, ^156^ For example, millions of neural progenitor cells undergo cell divisions and delamination to differentiate into specific functional regions during brain development, in which anoikis plays a crucial role in distinguishing the difference between the pathological detachment of progenitor cells and the normal delamination of daughter neuroblasts.^157^ Therefore, mechanisms involved in cells sensing and responding to the ECM changes, such as the anoikis, are important in promoting the organisms’ proper formation and growth.

In the maintenance of tissue homeostasis and functional repair, it is proved that anoikis was involved in the clearance of lumens in developing mammary glands^158^ and the involution of lactating mammary glands.^159^ Moreover, anoikis within luminal cells in the prostate epithelium occurs in conjunction with tissue repair programs during inflammatory damage and epithelial cell death processes caused by bacterial or viral infections.^160^ Considering the widely recognized notion that cell renewal occurs throughout the lifespan and cell signaling is influenced by the ECM and integrins,^161^ anoikis is considered to participate in the self‐renewal process of cellular life, as well as the body's inflammation and immune response processes,^162^ which in turn, maintains organism homeostasis and regulates tissue repair. However, in these life processes involving in anoikis, the role of ECM receptors and integrin response mechanisms has not yet been clearly reported and requires more attention in future researches.^163^

### 4.2 Anoikis in pathology

Anoikis is widely involved in pathological processes stimulated by external environmental interventions, particularly in the context of vascular function, nerve damage, tissue fibrosis, diabetes, fatty liver, tumor development, and CTCs metastasis.^164^, ^165^, ^166^, ^167^, ^168^ It is reported that the loss of functional contact with integrins can activate anoikis and impair vascular development, leading to chronic vascular diseases in diabetes, which is a major cause of mortality in diabetic patients.^169^ Anoikis is also implicated in the pathological remodeling of cardiovascular tissues, including detachment of cardiomyocytes in heart failure, endothelial denudation and plaque rupture in atherosclerosis, as well as smooth muscle cell loss in arterial aneurysms and varicose veins.^142^, ^144^, ^170^ Sater et al.^171^ have summarized the literature and concluded that anoikis hindered the recovery process of traumatic brain injury, exacerbated brain damage, and impaired synaptic plasticity and other central nervous system functions. However, the endocannabinoid‐metabolizing enzyme abhydrolase domain‐containing 4 has been shown to mediate anoikis specifically to protect the embryonic brain from the consequences of sporadic delamination errors and teratogenic insults.^157^ In the pathway regulating anoikis through integrins, arginine methylation of integrin alpha‐4 can prevent the development of fibrosis in alcohol‐related liver disease.^172^ The loss of osteocyte β3 integrin leads to abnormal cell morphology, as well as reduced bone mass and compromised biomechanical properties in weight‐bearing long bones of adult mice.^173^

Anoikis also plays a critical role in tumorigenesis and malignant transformation.^174^, ^175^ Therefore, it represents an important research focus with implications for drug treatment response, patient prognosis, and early cancer screening. Zhou and his colleagues^176^ found that the stabilization of XIAP (X‐linked inhibitor of apoptosis protein) by USP11 (an effective oncogenic factor) suppressed anoikis, promoting cancer development in breast epithelial cells, and increasing drug resistance in breast cancer cells. Similarly, inhibiting DOK2, a tumor suppressor in lung cancer, led to anoikis suppression and carboplatin resistance in ovarian cancer cells.^177^ However, whole‐genome sequencing analysis in gastric cancer proved that RHOA mutation in diffuse‐type tumors promoted anoikis escape, and the most disturbed pathway in gastric cancer happens to be adherens junction and focal adhesion. RHOA is a key participant, suggesting that anoikis maybe a promising target during the pathological process of the gastric cancer.^178^ Many studies also have identified anoikis‐related genes and established models that effectively predict the prognosis and immune landscape of various cancers, including ovarian serous cystadenocarcinoma,^179^ cutaneous melanoma,^180^ malignant pleural mesothelioma,^181^ and neuroblastoma.^162^

**FIGURE 5:**
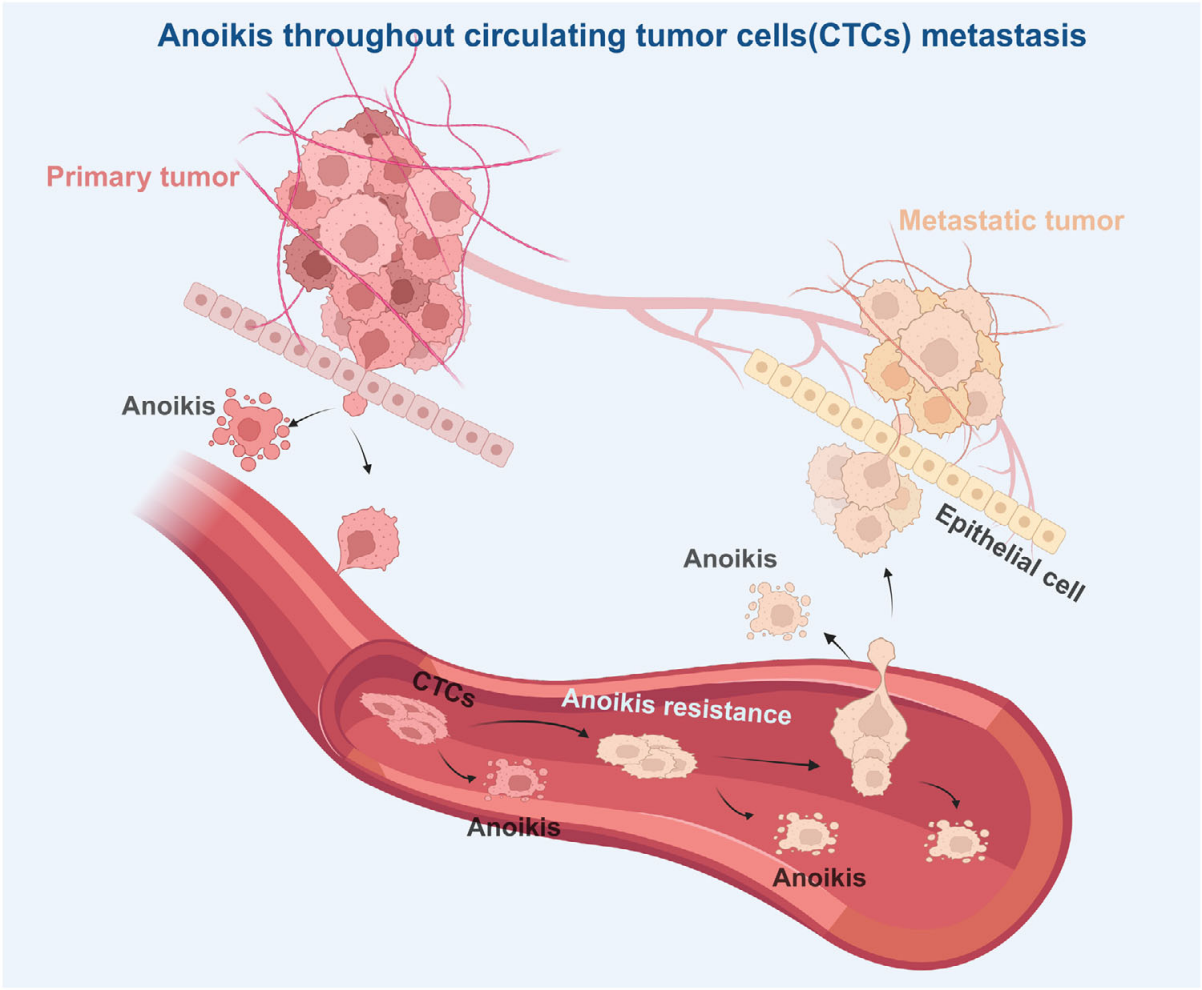
Anoikis is a necessary and ongoing barrier to tumor metastasis (CTC distant metastasis). During the process of tumor cells escaping from primary tumor and entering the blood, anoikis occurs continuously. Only the CTCs that maintains anoikis resistance can complete distant metastasis and colonization.

Appropriate ECM adhesion served as a barrier to tumor cell migration,^182^ and resistance to anoikis is often a necessary ongoing process in tumor metastasis (Figure 5). It is involved in the metastasis mechanisms of various cancer, including colorectal cancer,^183^ gastric cancer,^184^ prostate cancer,^185^ ovarian cancer,^59^ and breast cancer.^186^ It was reported that MYH9 can regulate CTNNB1 expression to induce anoikis resistance, promoting gastric cancer metastasis.^184^ CPT1A promoted anoikis resistance in esophageal squamous cell carcinoma^187^ and colorectal cancer^183^ via redox homeostasis, thereby facilitating distant metastasis. Mesenchymal stem cell‐derived IL‐8 could induce CXCR1/AKT signaling activation, thereby promoting anoikis resistance and pulmonary metastasis of osteosarcoma cells.^188^ Overcoming ECM nonpermissiveness and anoikis is often the first step for CTCs,^189^ which disseminate from the primary site through blood and lymphatic circulation, undergo processes of primary site detachment, entry into the bloodstream and lymphatic system, distant colonization, and survival.^118^ Interestingly, some studies have shown that microorganisms play a role in facilitating CTC metastasis.^190^ However, it remains unclear whether these microorganisms play a role in shaping CTC resistance against ECM detachment or if anoikis is involved in these mechanisms. Another representative event of tumor metastasis involving anoikis is the theory of peritoneal metastasis in epithelial ovarian cancer.^191^, ^192^ It is well known that ovarian cancer, especially high‐grade serous ovarian cancer (HGSOC), is accompanied by severe ascites,^193^ and one of the important theoretical hypotheses is that the tumor cells spread to the peritoneal cavity, which in turn acquires a stronger malignancy and proliferation capacity.^194^, ^195^ The survival of epithelial cells of tubal origin in the peritoneal cavity through the ovary has not been clearly explained.^196^ Anoikis has been identified as a driver of HGSOC metastasis. For example, CBX2 has been reported to promote the progression, metastasis, and chemoresistance of HGSOC through anoikis resistance.^197^ In addition, other pathways including Notch signaling pathway and autophagy regulator ULK1 have been identified as genetic features of anoikis escape in ovarian cancer cells.^198^ Gao's finding clearly articulated its mechanism by which HGSOC ascitic tumor cells characterized by integrin α5^high^ readily formed heterotypic spheroids with fibroblasts, which is associated with anoikis resistance in the ascites setting, ultimately both greatly contributed to its metastasis and early peritoneal spread.^199^, ^200^ Recent studies have recognized anoikis‐associated features as promising tools for assessing prognosis and predicting response to treatment of ovarian cancer.^201^, ^202^ Therefore, further attention and investigation in future research are warranted to shed light on these processes.

## 5 ANOIKIS IN THERAPEUTIC INTERVENTIONS

**FIGURE 6:**
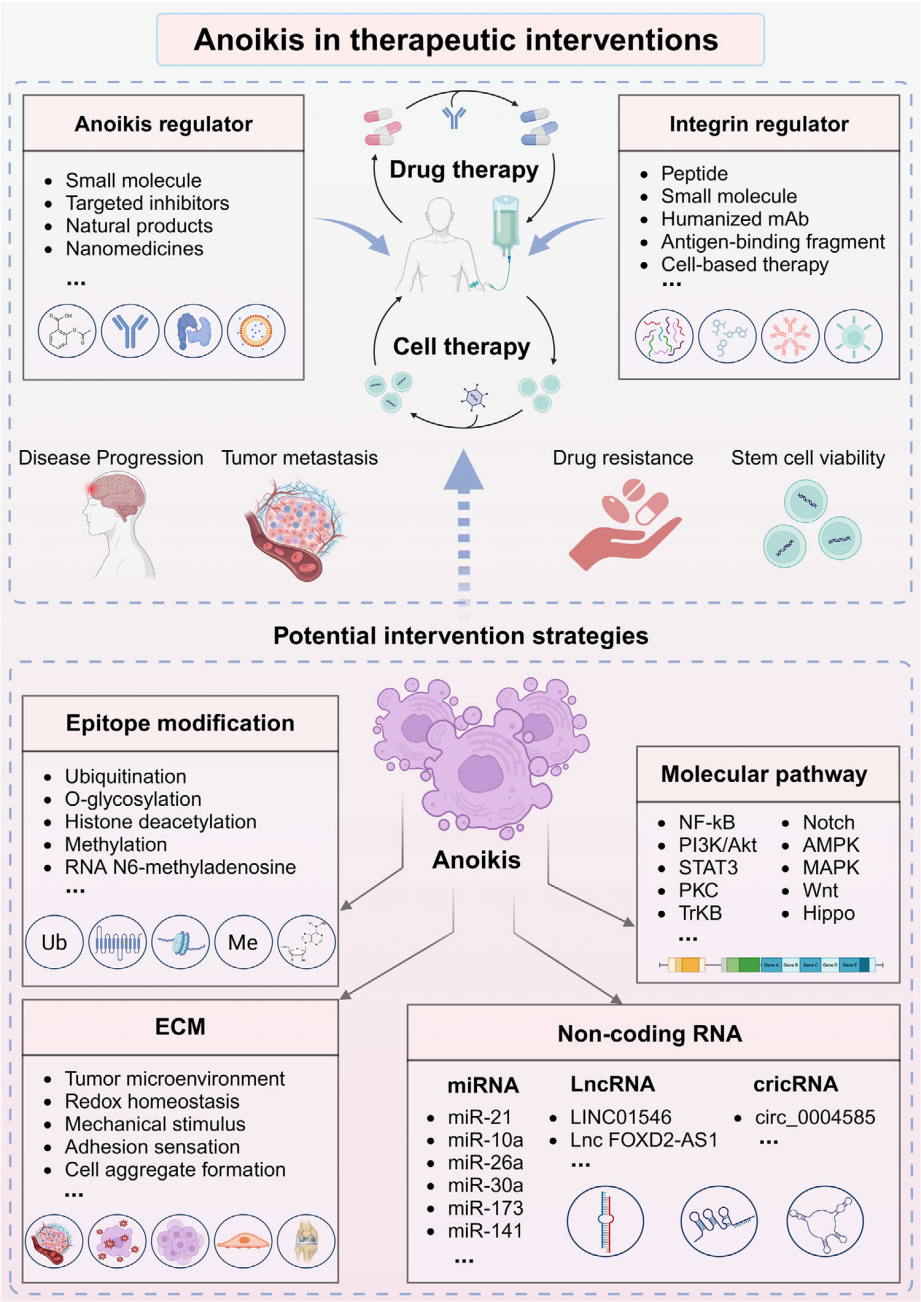
Anoikis in therapeutic interventions. Some regulatory drugs based on anoikis and integrins show potential to intervene in drug therapy and cell‐based therapy, which is helpful in overcoming disease progression, tumor metastasis, drug resistance, and stem cell survival issues. Additionally, there are several molecular pathways, noncoding RNAs, epigenetic modifications, and ECM regulation processes that can affect anoikis, which are potential intervention strategies for anoikis. ECM, extracellular matrix.

As mentioned above, the extensive roles of anoikis in individual cell fate and even in the physiopathology of the organism have been recognized, thus whether anoikis‐based regulatory are promising strategies for therapeutic interventions has attracted extensive research. Indeed, a number of studies have focused on the potential benefits of anoikis regulation for disease treatment,^203^, ^204^ and some studies on the mechanisms of anoikis regulation are being exploring,^204^, ^205^ which are potential directions for therapeutic intervention (Figure 6).

### 5.1 Intervention strategy of anoikis

Currently, anoikis is becoming a hallmark of cancer metastasis, so regulatory strategy based on anoikis resistance are extremely valuable in tumor research.^206^ NF‐kB,^207^ PI3K/Akt,^208^, ^209^ STAT3,^210^ PKC,^211^ TrkB,^212^ Notch,^213^ AMPK,^214^ MAPK,^63^ Wnt,^215^ and Hippo^216^ were proved to promote anoikis resistance. In addition, some non‐coding RNAs have been reported to be involved in the molecular process of anoikis.^185^, ^217^, ^218^, ^219^ Some epigenetic modification processes have also been suggested as possible anoikis regulatory strategies, including ubiquitination,^220^, ^221^ O‐glycosylation,^222^, ^223^ histone deacetylation,^224^ methylation,^225^, ^226^ RNA N6‐methyladenosine,^227^ and so on. It is worth mentioning that ECM regulation is also a possible direction, including ECM alterations,^228^ tumor microenvironment,^229^ redox homeostasis,^230^ cellular mechanosensation,^31^, ^231^ adhesion sensing,^189^ and colony formation^118^ processes, which are effective targets for the regulation of anoikis.

There is also a potential intervention strategy based on the integrin‐dependent mechanism of anoikis. Since integrins are required for ECM signaling to the cell, this means that integrins are naturally targets for the regulation of anoikis.^25^ As of June 2024, there have been over 140 clinical trials on integrins (https://www.clinicaltrials.gov/, https://www.clinicaltrialsregister.eu/, and http://www.chictr.org.cn using the search term “integrin”). Among them, the main chemotype of agents are small molecule, biologic (humanized mAb, antigen‐binding fragment), peptide, and so on, mainly targeting αLβ2, α4β7, α4β1, αIIbβ3, αvβ1, αvβ3, αvβ6, α5β1, αvβ8, αvβ5, α10β1, and αEβ7 dimers (Table 2). There are eight drugs targeting integrins have been marketed, but only Carotegrast is administered orally,^232^ while Efalizumab was withdrawn from the market in 2009 due to an increased risk of progressive multifocal leukoencephalopathy.^233^ These marketed drugs target the four integrins αIIbβ3, α4β7, α4β1 and αLβ2.^25^, ^53^ These clinical trials are suggesting the potential of integrin‐based regulatory for application in therapeutic interventions of disease, and likewise imply that integrin‐based regulatory strategies for anoikis may influence cell fate and thus the direction of disease therapy.

**TABLE 2:** Therapeutics based on integrin heterodimers.

Targeted integrins	Agents (chemotype)	Indication	Clinical trial number (study status)	Structure of integrin heterodimers
αLβ2	Lifitegrast (small molecule)^a^	Dry eye disease	NCT03686878 (completed), NCT03451396 (completed), NCT01636206 (completed)	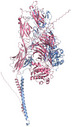
	Efalizumab (mAb)^b^	Plaque psoriasis	NCT00097240 (completed), NCT01079988 (completed), NCT00109252 (completed)	
	7HP349 (small molecule)	Solid tumors	NCT04508179 (completed)	
αIIbβ3	Tirofiban (small molecule)^a^	Acute coronary syndrome and thrombotic cardiovascular events	NCT01498003 (completed), NCT00566891 (completed), NCT00790387 (completed)	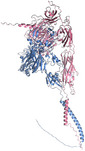
	Eptifibatide (small molecule)^a^	Acute coronary syndrome and thrombotic cardiovascular events	NCT01919723 (completed), NCT02925923 (completed), NCT00111566 (completed)	
	Abciximab (antigen‐binding fragment)^a^	Acute coronary syndrome and thrombotic cardiovascular events	NCT00379418 (completed), NCT00169819 (completed), NCT00271401 (completed)	
α4β7	Vedolizumab (mAb)^a^	Ulcerative colitis and Crohn's disease	NCT02788175 (completed), NCT05281614 (completed), NCT02862132 (completed)	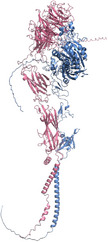
	Natalizumab (mAb)^a^	Multiple sclerosis and Crohn's disease	NCT00744679 (completed), NCT01884935 (completed), NCT00536120 (completed)	
	Carotegrast (small molecule)^a^	Moderate ulcerative colitis	Not available	
	PN‐943 (peptide)	Ulcerative colitis	NCT04504383 (completed)	
	MORF‐057 (small molecule)	Healthy volunteers	NCT04580745 (completed)	
	Etrolizumab (mAb)	Crohn disease	NCT02394028 (completed)	
	Firategrast/SB‐683699 (small molecule)	Multiple sclerosis	NCT00395317 (completed)	
α4β1	Natalizumab (mAb)^a^	Multiple sclerosis and Crohn's disease	NCT00744679 (completed), NCT01884935 (completed), NCT00536120 (completed)	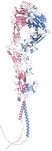
	Carotegrast (small molecule)^a^	Moderate ulcerative colitis	Not available	
	7HP349 (small molecule)	Solid tumors	NCT04508179 (completed)	
	Firategrast/SB‐683699 (small molecule)	Multiple sclerosis	NCT00395317 (completed)	
α5β1	JSM‐6427 (small molecule)	Age‐related macular degeneration	NCT00536016 (completed)	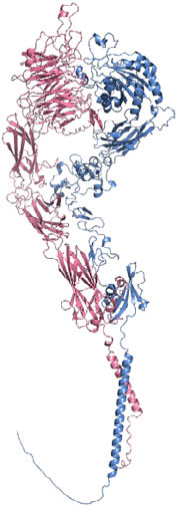
	AXT107 (peptide)	Diabetic macular edema	NCT04697758 (terminated)	
		Age‐related macular degeneration	NCT04746963 (terminated)	
	Volociximab (mAb)	Age‐related macular degeneration	NCT00782093 (completed)	
		Melanoma	NCT00099970 (completed)	
		Pancreatic cancer	NCT00401570 (completed)	
	PF‐04605412 (mAb)	Nonhematologic malignancies	NCT00915278 (terminated)	
	Plasmid AMEP (gene therapy)	Melanoma	2011‐005538‐20 (prematurely ended)	
αvβ5	Intetumumab (mAb)	Prostatic neoplasms	NCT00537381 (completed)	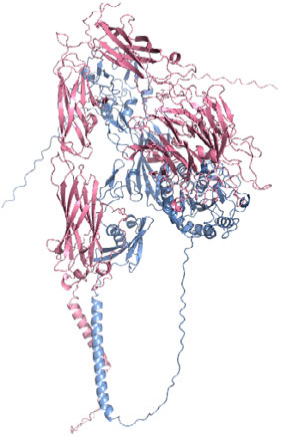
		Solid tumors	NCT00888043 (completed)	
		Melanoma	NCT00246012 (completed)	
	Abituzumab (mAb)	Colorectal and ovarian cancer patients with liver metastases	NCT00848510 (completed)	
		Prostate cancer	NCT00958477 (completed), NCT01360840 (completed)	
αvβ6	IDL‐2965 (small molecule)	Idiopathic pulmonary fibrosis and nonalcoholic steatohepatitis	NCT03949530 (terminated)	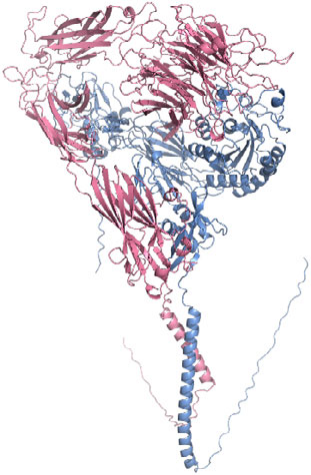
	PLN‐74809 (small molecule)	Idiopathic pulmonary fibrosis	NCT04072315 (completed), NCT04396756 (completed)	
		Primary sclerosing cholangitis	NCT04480840 (completed)	
	JSM‐6427 (small molecule)	Age‐related macular degeneration	NCT00536016 (completed)	
	Intetumumab (mAb)	Prostatic neoplasms	NCT00537381 (completed)	
		Solid tumors	NCT00888043 (completed)	
		Melanoma	NCT00246012 (completed)	
	Abituzumab (mAb)	Colorectal and ovarian cancer patients with liver metastases	NCT00848510 (completed)	
		Prostate cancer	NCT00958477 (completed), NCT01360840 (completed)	
αEβ7	Etrolizumab (mAb)	Crohn disease	NCT02394028 (completed)	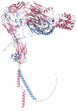
α10β1	XSTEM‐VLU (Cell‐based therapy)	Venous leg ulcer	NCT05549609 (recruiting)	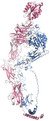
αvβ1	IDL‐2965 (small molecule)	Idiopathic pulmonary fibrosis and nonalcoholic steatohepatitis	NCT03949530 (terminated)	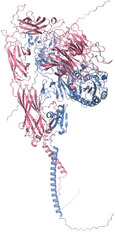
	PLN‐74809 (small molecule)	Idiopathic pulmonary fibrosis	NCT04072315 (completed), NCT04396756 (completed)	
		Primary sclerosing cholangitis	NCT04480840 (completed)	
	PLN‐1474 (small molecule)	End‐stage liver fibrosis in nonalcoholic steatohepatitis	Not available	
	Intetumumab (mAb)	Prostatic neoplasms	NCT00537381 (completed)	
		Solid tumors	NCT00888043 (completed)	
		Melanoma	NCT00246012 (completed)	
	Abituzumab (mAb)	Colorectal and ovarian cancer patients with liver metastases	NCT00848510 (completed)	
		Prostate cancer	NCT00958477 (completed), NCT01360840 (completed)	
αvβ8	JSM‐6427 (small molecule)	Age‐related macular degeneration	NCT00536016 (completed)	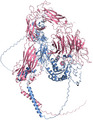
	Abituzumab (mAb)	Colorectal and ovarian cancer patients with liver metastases	NCT00848510 (completed)	
		Prostate cancer	NCT00958477 (completed), NCT01360840 (completed)	
αvβ3	IDL‐2965 (small molecule)	Idiopathic pulmonary fibrosis and nonalcoholic steatohepatitis	NCT03949530 (terminated)	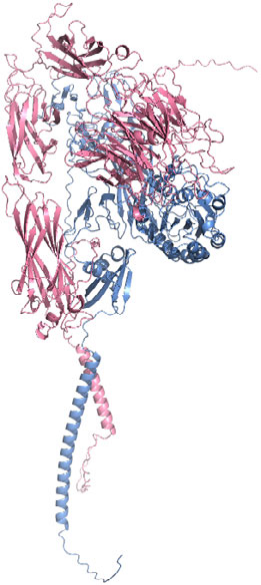
	ProAgio (peptide)	Pancreatic cancer and solid tumor malignancies	NCT05085548 (recruiting)	
		Pancreatic ductal adenocarcinoma	NCT06182072 (recruiting)	
	AXT107 (peptide)	Diabetic macular edema	NCT04697758 (terminated)	
		Age‐related macular degeneration	NCT04746963 (terminated)	
	Etaracizumab/MEDI522 (mAb)	Colorectal cancer or other solid tumors	NCT00284817 (completed)	
		Plaque psoriasis	NCT00192517 (completed)	
		Rheumatoid arthritis	NCT00069017 (completed)	
	Intetumumab (mAb)	Prostatic neoplasms	NCT00537381 (completed)	
		Solid tumors	NCT00888043 (completed)	
		Melanoma	NCT00246012 (completed)	
	Plasmid AMEP (gene therapy)	Melanoma	2011‐005538‐20 (prematurely ended)	
	Abituzumab (mAb)	Colorectal cancer	NCT01008475 (completed)	
		Solid tumor	NCT01327313 (completed)	
		Colorectal and ovarian cancer patients with liver metastases	NCT00848510 (completed)	

Superscript “a” refers to the marketed drugs, and “b” refers to the drug that have been marketed but withdrawn in 2009. The clinical trials of integrins were obtained from ClinicalTrials.gov (https://www.clinicaltrials.gov/) and EudraCT (https://www.clinicaltrialsregister.eu/ctr‐search). AlphaFold 3^234^ (https://golgi.sandbox.google.com/) predicted the structure of integrin heterodimers.

In summary, anoikis, as an integral aspect of cell fate, significantly influences the physicochemical sensation of cells. Consequently, alterations happened in the anoikis process can affect the effectiveness of various therapeutic strategies in the clinic. Targeting anoikis through molecular regulation and ECM signaling offers promising approaches to overcoming therapeutic resistance and guides the development of more effective targeted therapies for diseases.

### 5.2 Anoikis and drug development

Anoikis, as a type of apoptosis influenced by the ECM, is not only a risk factor for the development, progression, and metastasis of many diseases,^9^, ^235^ but also contributes to drug or therapeutic resistance.^40^ Therefore, a detailed understanding of the molecular pathways and regulatory mechanisms of anoikis is essential for designing effective targeted therapeutic strategies and addressing drug resistance.

Tajbakhsh et al.^236^ have argued that anoikis resistance is responsible for tumor development, therefore drug development based on anoikis‐related molecular pathways are extremely important for the treatment of triple‐negative breast cancer. These include DSF, nanoencapsulated doxorubicin, berberine, salinomycin, tubeimoside V, 5‐azacytidine, synthesized flavonoid derivative GL‐V9 and HPW‐RX40, and other compounds have been recognized as potential agents for regulating anoikis resistance in breast cancer cells.^236^ In studies of HGSOC precancerous lesions, norepinephrine has been found to instigate anchorage independence and micrometastasis of preneoplastic lesions from the fallopian tube epithelium to the ovary, which is largely accomplished through β‐adrenergic receptor‐dependent anoikis resistance, and the use of the β‐adrenergic receptor blocker propranolol eliminates the globule formation and cell viability conferred by norepinephrine.^237^

On the other hand, the effects of ECM alterations on cell fate as well as drug sensitivity have long been of interest in in vitro studies.^228^ It has been found that SKOV3 cells cultured in an ultra‐low apposition system exhibit anoikis resistance. The key factor may be the lncRNA HOX transcript antisense RNA (HOTAIR), which may contribute to anoikis resistance by recruiting EZH2 and affecting H3K27 methylation to enhance sphere formation in ovarian cancer cells. This may be one of the mechanisms of ovarian cancer metastasis and chemoresistance, so the anoikis regulatory pathway mechanism based on HOTAIR provides a possible pathway to target tumor metastasis and chemoresistance.^226^ Some studies have investigated the mechanisms underlying anoikis resistance in the suspension culture of ovarian cancer spheroids and demonstrated that modifications in the mitochondrial activity was induced by visfatin, suggesting that visfatin is a potential new therapeutic target for the treatment of peritoneal disseminated ovarian cancer.^102^ Wang et al. developed an ECM deprivation system (EDS) to regulate anoikis. Based on previous studies, fibronectin was found to have a direct regulatory effect on ECM‐mediated anoikis resistance and cell adhesion‐mediated drug resistance.^238^ Therefore, the EDS constructed based on FN‐targeted self‐assembling peptides could reverse anoikis resistance by blocking FN signaling, while improving chemotherapeutic drug sensitivity, which is an innovative way of anoikis regulation and provides new ideas for anoikis‐based drug sensitivity regulation.^228^ In vivo studies also have similar findings. For example, during the process of tumor peritoneal metastasis, individual cancer cell suspended in peritoneal fluid can aggregate to form multicellular spheroids. This cellular arrangement confers resistance to anoikis, apoptosis, and chemotherapeutic agents.^239^

Therefore, based on the clear link between anoikis and tumor metastasis as well as drug resistance, the molecular pathways of anoikis are considered to be highly promising therapeutic and intervention targets. For instance, HMGA1 was found to promote anoikis resistance in pancreatic cancer cells through PI3‐K/Akt‐dependent mechanism, whereas inhibition of PI3‐K/Akt by the small molecule inhibitor LY294002 or dominant‐negative Akt could reverse this cell fate. Given the association between anoikis resistance and the PI3‐K/Akt pathway, HMGA1 was regarded as a potential therapeutic target for pancreatic cancer.^240^ Claudin‐1 has been shown to play a role in promoting metastasis and anoikis resistance in colorectal cancer.^241^, ^242^, ^243^ In addition, Claudin‐1 has also been implicated in tumor stemness and chemoresistance‐related signaling pathways and promotes stemness and chemoresistance by increasing EPHA2 expression, downstream AKT signal transduction, and CD44 expression. Therefore, Claudin‐1 is considered as a highly promising target for drug intervention.^244^ For chemoresistance in prostate cancer treatment, the epithelial‐mesenchymal transition process mediated by TGF‐β confers stemness, which in turn promotes tumor migration and chemoresistance through anoikis resistance.^245^ DZ‐50 was found to induce anoikis and show certain therapeutic effects.^246^ Moreover, tumor‐infiltrating adipose stem cells promoted glycolysis and anoikis resistance in colorectal cancer cells, and ultimately resulted in peritoneal metastasis through the TGF‐β1/SMAD3/ANGPTL4 axis. Dual targeting of TGF‐β signaling and ANGPTL4 may be a viable therapeutic strategy for peritoneal metastasis of colorectal cancer.^247^ These suggest that more mechanisms about anoikis regulatory have yet to be explored for application in drug therapy.

### 5.3 Anoikis and cell‐based therapies

Anoikis has demonstrated significant potential in the field emerging cell‐based therapies. Its influence on maintaining cell stemness for oncology research and its impact on the viability of engineered stem cells in therapeutic applications are particularly.

For tumor stem cells, anoikis resistance of tumor cells is an important part of their increased stemness.^46^ Fibrinolytic factor can promote the survival and growth of tumor cells by interfering with cell anoikis resistance to provide more survival opportunities for the cells.^248^ TrkB has also been reported to enhance cell metastasis by increasing anoikis resistance,^249^ and interestingly, it also promotes stemness in hepatocellular carcinoma cells by increasing the stability of DJ‐1, aiding in tumor progression.^250^ Liu et al.^251^ established a therapeutic strategy in triple‐negative breast cancer by targeting the tumor stemness. They found Tetrandrine, a natural plant alkaloid, can effectively inhibit breast cancer stem cell characteristics by promoting anoikis, and is therefore considered a potential antitumor drug.^251^ RhoC has also been proved to regulate a variety of cellular phenotypes including metastasis and anoikis resistance, and thereby conferring plasticity to help the cells gain more viability. Thus, RhoC is considered as a cancer stem cell therapeutic target.^252^

In addition, for engineered stem cells such as ESCs and iPSC, they have many problems such as difficulties in in vitro culture, expansion and preservation,^253^, ^254^ and the maintenance of their activity for therapeutic use remains an insurmountable problem,^255^ which severely limits their use in many cell‐based therapies. Considering the adhesion‐dependent properties required for most stem cells, overcoming anoikis to help cells gain more survival activity becomes a promising direction.^256^ One of the important problems in cell transplantation is the degrafting‐induced anoikis. Frisco‐Cabanos et al.^140^ have found that water‐soluble molecules can protect suspension cells from anoikis by integrin‐mediated and thus increase cell survival. For some neurodegenerative diseases, the therapeutic strategy of cell transplantation has been fraught with difficulties centered on improving cell survival after transplantation, and anoikis is likely to be an important cause of cell death after neuronal transplantation to the brain.^257^ Of course, some gel systems or adhesion matrices can help to improve cell survival.^258^ Schaschkow et al.^167^ reported that a hydroxypropyl methylcellulose hydrogel can help to reduce anoikis and promote blood vessel formation in islet transplantation, which is extremely valuable for glycemic control in diabetes. hESC‐CMs transplantation in cardiac research showed that cotransplantation of biocompatible materials or hydrogels with hESC‐CMs could promote cellular anoikis resistance, which in turn increased hESC‐CMs’ survival after transplantation and promote the recovery of cardiac function.^144^ All these are as Michel^170^ emphasized that anoikis is extremely helpful in cardiovascular system diseases for the pathological remission. Similarly, hESC‐RPE has been verified to undergo anoikis during therapy due to factors like ECM segregation and serum deprivation. Therefore, hESC‐RPE cells that overcome anoikis hold promise as an in vitro model for stem cell therapy.^132^

In summary, the application potential of anoikis in cell‐based therapies is still far from being explored. For solving the problem of tumor cell stemness, anoikis‐related signaling remains to be studied as an important therapeutic target. On the other hand, in the engineering application of stem cells, it is reasonable to solve the pre‐ and post‐treatment survival of cells based on anoikis regulation. Therefore, anoikis has great potential for research in stem cell differentiation, cell transplantation and other cell‐based therapies.

## 6 CONCLUSION AND PROSPECTS

Since the establishment of the concept of apoptosis in 1972,^259^ research on cell death has never ceased, making it a crucial area of study in understanding cell fate.^260^, ^261^ The discovery and research of emerging death modes, including anoikis,^7^ disulfidptosis,^262^ cuproptosis,^263^ and paraptosis,^264^ are advancing human understanding and application of cell biology. The primary tool for these investigations has been the in vitro cell model. As a fundamental model in biomedical research, cell models have been extensively utilized to simulate the physiological and pathological environments in the body.^265^ However, the significant differences between these traditional research models and the in vivo microenvironment have raised concerns among researchers in the fields of developmental biology, pathology, and pharmacology.^266^ In particular, with the emergence of genetically engineered animal models^267^ and organoids,^268^, ^269^ conventional 2D cell culture in vitro can no longer meet the demands of rapidly advancing research.^270^ Consequently, cell coculture systems,^271^ 3D culture systems,^272^ and cell culture protocols based on biomaterials^273^ have been developed to address a crucial issue—creating an environment that closely resembles the in vivo growth conditions. This includes the construction of ECM suitable for cell survival, which undoubtedly contributes to recapitulating the programmed cell death states.^274^

Traditional cell models include primary cell models, which are derived from their original tissue but no longer retain the growth state of the primary cells. These models undergo significant genomics^275^, ^276^ and epigenomics,^277^ regulations, and the impact of the ECM on cell adhesion, migration, and cell fate (including cell death), are more immediate and direct.^93^, ^278^ Anoikis, as an important programmed cell death mode regulated by the ECM, determines the fate of cells from the moment they detach from their original environment and undergo in vitro screening and culture. It is for this reason that only cells capable of adapting to the new environment have the potential to survive. This implies that the initial round of anoikis phenotype regulation occurs at the onset of cell detachment and continues until the cells establish a new adhesive growth system.^118^, ^279^ Therefore, we currently cannot fully address whether the research based on these newly established cell models with adaptive changes reflects the true in vivo behavioral and genomic states. However, when focusing on the study of the cell death fate process, it is essential to maintain as much consistency as possible with the in vivo environment in the anoikis process regulated by the ECM. This may have unexpected value in recapitulating cell behavioral states and micro‐scale genetic information.

For engineered cells such as ESCs, iPSCs, and so on, their research direction is relatively pure. Researchers often focus more on their functional extensions rather than their original survival states.^280^ Due to their high pluripotency and strong differentiation potential,^85^ these cells exhibit significant adaptability and changes. Therefore, researchers are more concerned with finding the most suitable growth environment for these cells rather than the original one. Similarly, this applies to the transformation research of functional derivative cell lines.^281^ Currently, there are significant limitations in the culture, differentiation, and storage of stem cells,^282^ including challenges in proliferation,^283^ efficiency of differentiation,^284^ and interference in cryopreservation.^285^ A core issue being addressed is the reduction of cell death, especially ECM‐mediated anoikis. The strategies employed are imaginative and include genomic regulation,^286^ upgrading of culture media,^287^ and changes in matrix conditions.^288^ Researchers have also developed various biomaterials to assist in the growth and proliferation of stem cells, including specialized material surfaces,^289^ hydrogel systems,^290^ and nanotopography,^291^ all of which have been reported to varying degrees to resist anoikis. Although some studies suggest that pathways such as ferroptosis also participate in the process of stem cell detachment‐induced death,^292^ the inhibition of anoikis‐mediated stem cell growth and differentiation remains a major potential direction.

Of course, anoikis also participates in a wide range of physiological and pathological processes. Since the development of the fertilized egg, the fate of death has been engraved in the genetic code of every cell.^293^ In the early stages of embryonic development, organ formation begins, accompanied by the initiation of distal migration. And the exact cell fate during this period remains unclear, which could involve survival through resistance to anoikis or adaptive survival of anoikis‐resistant cells. In the process of cellular aging, cell death often accompanies cell detachment and clearance from the body.^294^ Anoikis is an important component of cellular senescence and involved in various pathological processes, including diabetes, cardiovascular diseases, tumor metastasis and more.^79^ Whether targeting anoikis is a potential therapeutic target is still unclear.^295^ However, this does not mean that anoikis cannot be involved in disease treatment interventions.

Studies of anoikis‐based therapeutic interventions in tumor metastasis, drug resistance have been reported,^184^, ^296^ and more anoikis agents are under intense experimental investigation.^206^ Some drugs that have been in clinical use for a long time have shown to regulate anoikis and thus affect cell fate.^237^ Natural products, polysaccharide,^297^ small molecules such as A‐1210477,^298^ and nanomedicines such as human serum albumin combined with doxorubicin^299^ have also shown inhibitory effects on anoikis resistance. The agents targeted in integrin have also been explored in numerous clinical trials, and they are all highly promising agents for anoikis intervention. Thus anoikis‐based regulation is certainly helpful in drug therapy. On the other hand, cell therapies based on stem cells are currently considered to have significant development potential in various fields such as tissue repair, Alzheimer's disease, and cancer.^300^, ^301^, ^302^ Preliminary results have been achieved with stem cell and cell transplantation therapies that assist in resisting anoikis.^140^ In oncology research, chimeric antigen receptor‐T cell therapies,^303^ T cell receptor‐T cell therapies,^304^ and tumor‐infiltrating lymphocyte therapies,^305^ which are currently receiving considerable attention, may also need to consider the interfering factors of anoikis in cellular fate. Finally, cell–cell interactions^118^, ^306^ and microbial signaling,^307^ which are also part of extracellular signals, may regulate the process of anoikis. Indeed, the ultimate goal is to improve the patient benefit of cell‐based therapy by increasing the activity of various types of engineered stem cells through various physicochemical means. All of these indicate that anoikis acts as a hidden player in the regulation of cell fate and physiopathology process. Exploring the interplay between external signals and micro‐mechanisms could suggest valuable research directions for therapeutic interventions.

In summary, anoikis is a distinct form of apoptosis that cannot be simply categorized as traditional apoptosis due to its unique initiation process, which including extracellular physical and chemical stimuli triggering changes in the ECM. Anoikis plays a broader role in cell fate, as well as physiological and pathological regulatory processes, and presents new challenges and opportunities for the self‐detection of engineered cell states and cutting‐edge applications. Therefore, the therapeutic potential of targeting anoikis in drug therapies and cell‐based treatments attracts focused attention. Further studies are required to identify the genetic regulators, chemicals, and novel culture materials that can specifically target the anoikis. Such advancements could address the challenges proposed in this review, enhance the establishment of in vitro cell models and stem cells induction, and ultimately facilitate the application of anoikis‐based interventional therapies in the clinic for the benefit of patients.

## AUTHOR CONTRIBUTIONS

Jie Mei, Lei‐Yun Wang, Ji‐Ye Yin and Peng‐Yuan Wang conceived the manuscript. Jie Mei, Xue‐Yao Jiang, Hui‐Xiang Tian, and Jia‐Nan Song primarily searched for the papers and made the outline. Jie Mei, Hui‐Xiang Tian, and Jia‐Nan Song wrote the initial draft; Jie Mei, Ding‐Chao Rong, LuozixianWang, Jia‐Nan Song, and Lei‐Yun Wang polished the manuscript. Jie Mei, Hui‐Xiang Tian, Ding‐Chao Rong, Xue‐Yao Jiang, and Yuan‐Shen Chen designed and drew the figures. Xue‐Yao Jiang and Hui‐Xiang Tian checked all references and formatting. Ji‐Ye Yin, Peng‐Yuan Wang, Raymond C. B.Wong, Cheng‐Xian Guo, and Lian‐Sheng Wang reviewed the manuscript and provided suggestions for revision. All authors revised and contributed to the final version of the manuscript.

## CONFLICT OF INTEREST STATEMENT

The authors declare no conflict of interest.

## ETHICS STATEMENT

Not applicable.

## Data Availability

Not applicable.
